# Are different station formats assessing different dimensions in multiple mini-interviews? Findings from the Canadian integrated French multiple mini-interviews

**DOI:** 10.1186/s12909-022-03681-4

**Published:** 2022-08-12

**Authors:** Jean-Michel Leduc, Sébastien Béland, Jean-Sébastien Renaud, Philippe Bégin, Robert Gagnon, Annie Ouellet, Christian Bourdy, Nathalie Loye

**Affiliations:** 1 Centre de recherche du Centre intégré universitaire de santé et de services sociaux du Nord-de-l’Île-de-Montréal, Hôpital du Sacré-Cœur de Montréal, 5400 boul. Gouin ouest, Montréal, QC H4J 1C5 Canada; 2 Department of Microbiology, Infectious Diseases and Immunology, Faculty of Medicine, Université de Montréal, 2900 boul. Edouard-Montpetit, Montréal, QC H3T 1J4 Canada; 3 Department of Education Administration and Foundations, Faculty of Education Sciences, Université de Montréal, 90, avenue Vincent-D’Indy, Montréal, QC H2V 2S9 Canada; 4 Department of Family Medicine and Emergency Medicine, Office of Education and Professional Development, Faculty of Medicine, Université Laval, 1050 Avenue de la Médecine, Quebec, QC G1V 0A6 Canada; 5 Department of Medicine, Faculty of Medicine, Université de Montréal, 2900 boul. Edouard-Montpetit, Montréal, QC H3T 1J4 Canada; 6 Office of Assessment and Evaluation, Faculty of Medicine, Université de Montréal, 2900 boul. Edouard-Montpetit, Montréal, QC H3T 1J4 Canada; 7 Department of Obstetrics and Gynecology, Faculty of Medicine and Health Sciences, Université de Sherbrooke, 3001 12 Ave N Immeuble X1, Sherbrooke, QC J1H 5N4 Canada; 8 Department of Family Medicine and Emergency Medicine, Faculty of Medicine, Université de Montréal, 2900 boul. Edouard-Montpetit, Montréal, QC H3T 1J4 Canada

**Keywords:** Selection, Admission, Undergraduate Medical Education, Multiple Mini-Interviews, Validity, Reliability

## Abstract

**Background:**

Multiple mini-interviews (MMI) are used to assess non-academic attributes for selection in medicine and other healthcare professions. It remains unclear if different MMI station formats (discussions, role-plays, collaboration) assess different dimensions.

**Methods:**

Based on station formats of the 2018 and 2019 Integrated French MMI (IFMMI), which comprised five discussions, three role-plays and two collaboration stations, the authors performed confirmatory factor analysis (CFA) using the lavaan 0.6-5 R package and compared a one-factor solution to a three-factor solution for scores of the 2018 (*n* = 1438) and 2019 (*n* = 1440) cohorts of the IFMMI across three medical schools in Quebec, Canada.

**Results:**

The three-factor solution was retained, with discussions, role-plays and collaboration stations all loading adequately with their scores. Furthermore, all three factors had moderate-to-high covariance (range 0.44 to 0.64). The model fit was also excellent with a Comparative fit index (CFI) of 0.983 (good if > 0.9), a Tucker Lewis index of 0.976 (good if > 0.95), a Standardized Root Mean Square Residual of 0.021 (good if < .08) and a Root Mean Square Error of 0.023 (good if < 0.08) for 2018 and similar results for 2019. In comparison, the single factor solution presented a lower fit (CFI = 0.819, TLI = 0.767, SRMR = 0.049 and RMSEA = 0.070).

**Conclusions:**

The IFMMI assessed three dimensions that were related to stations formats, a finding that was consistent across two cohorts. This suggests that different station formats may be assessing different skills, and has implications for the choice of appropriate reliability metrics and the interpretation of scores. Further studies should try to characterize the underlying constructs associated with each station format and look for differential predictive validity according to these formats.

## Background

Multiple mini-interviews (MMI) are increasingly used worldwide as tools to assess non-academic attributes for selection in the healthcare professions [1]. They were first implemented at McMaster for medical school selection in 2002 [2] and were designed to reduce the context specificity observed with traditional interviews. They generally consist of a series of short structured or semi-structured interviews or role-plays with actors [3] and, depending on their implementation parameters, may show conceptual overlap with Assessment Centers (AC), which also have multiple components aimed to assess specific behaviors [4]. Since MMIs are usually a very high-stake assessment tool, evidence for their validity is of the utmost importance. According to Kane [5], validity should be conceived as a validation process, rather than a concept to be broken down into many forms (e.g. face validity, construct validity, predictive validity, etc.). The goal is to provide evidence related to 1) how the instrument was developed (content and scoring), 2) the accuracy or stability of the scores obtained (reliability and generalizability), 3) the constructs that are assessed and possible sources of unwanted variance (extrapolation) and 4) the credibility and implications of the decisions that flow from the process [6, 7]. A recent review suggested that more data was needed regarding the construct validity evidence of MMI [4], which consists mostly of extrapolation validity evidence in Kane’s framework.

What exactly is assessed by MMIs remain elusive and likely vary depending on the implementation parameters and actual content [8]. In some instances, authors have suggested it could be “problem evaluation” [9] or more recently “adaptability” or “ability to identify criteria” [10]. A fairly consistent finding is that MMI scores are uncorrelated or inversely correlated to GPA or other measures of previous academic performance [11–13]. Positive associations were found between MMI scores and OSCE scores in medical school [12, 14–16], clerkship rotation evaluations [16–18], and in some contexts with exam scores [15]. Two multicenter studies have found correlations between MMI that were developed and implemented by institutions independently [10, 19], suggesting some overlap between the constructs assessed in various settings. Moreover, a recent systematic review of personal domains assessed in MMI demonstrated that a few personal characteristics, such as communication skills and collaboration, were included in the design of most MMIs described in the literature [20].

In various settings, authors have tried to study the dimensionality of MMIs, i.e. the number of latent variables or constructs that are measured, with mixed results. For example, exploratory factor analysis (EFA) studies by Lemay et al. [21] and by Cox et al. [22] identified that each of their MMI station formed a factor and was likely to assess a different dimension. An EFA study in veterinary medicine on a 5-station MMI (semi-structured interviews with behavioural questions) ended up with a 3-factor solution (i.e. three dimensions) labelled “moral and ethical values”, “interpersonal ability” and “academic ability”, which also combined applicant’s age and GPA. More recently, an Australian study suggested that MMI in different Australian institutions were unidimensional [10].

MMIs for selection in medicine use a vast array of station formats and, arguably, applicants will need to rely on a different set of skills to perform in these various types of stations. In the AC literature, even with distinct components, most of the performance difference observed will vary according to the simulation exercise rather than any underlying pre-specified construct [23]. From a theoretical perspective, station formats can be considered one of the “building blocks” of an MMI modular design process that will likely provide different levels of contextualization and stimulus presentation consistency [24]. For example, scripted role-plays will usually provide a very high and detailed contextualization that could mirror social interaction in “real-life”, just like simulated patients [25], whereas discussion stations, often less contextualized and more “open-ended”, are likely to require more reflection and argumentation skills. Therefore, exploring how different station formats (e.g. discussion, role-plays, etc.) contribute to scoring is highly relevant since it is a design choice over which admission committees have full control. Indeed, if all MMI stations seem to be assessing the same dimension, then the stations within a given MMI are most likely interchangeable and could be chosen according to other factors such as ease of implementation or cost. For example, in our experience, role-plays are usually more complex and time-consuming to plan and may add some inconsistencies related to the actor’s performance. On the other hand, if station formats are assessing different dimensions, it then becomes important to assess if they all bring relevant information to the process and explore the use of subscores to inform admission decisions. Furthermore, reliability issues can emerge, since some dimensions will be assessed by fewer items. In a recent retrospective analysis looking at the psychometric properties of role-play and interview stations in the Integrated French Multiple Mini-Interviews (IFMMI), Renaud et al. showed that factor models considering these two station formats as two dimensions could best explain the structure of the test [17]. This analysis, however, did not include more recent iterations of the IFMMI where a third type of station was added (collaboration).

Therefore, the goal of this study was to see if, in our context, stations with three different formats could possibly assess different underlying dimensions. The IFMMI is a collaborative effort between the three French-speaking medical schools in Quebec (Canada). Each year, approximately 1600 applicants are assessed over a weekend in four interview centers located in Montreal, Quebec City, Sherbrooke and Moncton. The interview score is then shared between the three medical schools, so that candidates applying to more than one institution need to do the interviews only once. Each institution then uses the global interview score according to their own selection criteria. Overall, in 2018 and 2019, the weight given to the IFMMI was about 50% of the final score before ranking for admission offers, the other 50% being given to the R score (academic performance score) [26]. In recent years, the IFMMI relied on a mix of discussion stations, role-plays and collaborative stations. It has already been found to show reliable scores [17, 27] and some predictive validity with clerkship rotation performance [17, 18]. Thus, the present study is part of a validation process which aims to appraise the dimensions that are evaluated by the IFMMI on the basis of their station format. Drawing on recent work done on two types of stations [17], we postulated that each station format would assess a different dimensions and that, therefore, a three-dimensional structure would provide a better fit for our MMI results than a unidimensional structure.

## Methods

In 2018 and 2019, the IFMMI consisted of a 10-station circuit, each of 7-minute duration, including five stations with semi-structured discussions with an assessor, three role-play stations with actors, and two collaborative stations where candidates were asked to complete a task while working in teams of two. Examples of design and layouts are provided in Table 1. Although station content was different between the 2 years, the station formats remained the same. Grading in each station was made using a single 6-point Likert-scale (A to F) referring to a station-specific scoring grid with general anchors (A-Excellent, B-Very Good, C-Good, D-Borderline, E-Obvious gaps, F-Insufficient) and then converted to a numerical value between 0 and 100 using a previously-validated asymmetric scale (A = 100, B = 86.7, C = 69.5, D = 51.2, E = 29.3, F = 0) [28]. Stations 2 & 3 (collaborative stations) had the same scoring grid. Before being computed into a final score, individual station scores were also normalized by rater and by station, to account for rater stringency and station difficulty. This study received IRB approval from the Comité d’éthique sur la recherche en santé at Université de Montréal (Certificate 17-038-CPER-D). All methods were carried out in accordance with relevant guidelines and regulations.

**Table 1:** Examples of designs according to station formats

Station format	Example of design (all are 7-minute stations)
Discussion	Candidates must give their opinion on the role of artificial intelligence in medicine and healthcare
Role-play	Scenario: While on vacation in a hostel, you meet someone who seems to have an alcohol problem (played by an actor). The candidate must then interact with the actor to better understand the situation.
Collaborative station	Candidates must collaborate together to build something with the provided material (e.g. blocks, cards) by using instructions given to each candidate. At the end, they are asked to reflect on their interaction with the other participant.

Based on station formats (discussion, role-play and collaboration), we performed a three-factor confirmatory factor analysis (CFA) using R 4.0.3 (R Core Team, 2020) and the Lavaan package (v0.6-5, Rosseel, 2012). We then compared it to a single-factor model to see if a model built according to station formats would provide a better fit. We report the standardized factor loadings and model covariances in addition to four fit indexes: Comparative Fit Index (CFI) where a good fit occurs when CFI ≥ .90, Tucker Lewis index (TLI) where a good fit is TLI ≥ .95, Standardized Root Mean Square Residual (SRMR) where a good fit is SRMR < .08 and Root Mean Square Error of Approximation (RMSEA) where a good fit is also when RMSEA < .08 [29].

To assess invariance between years, we used a multiple group CFA relying on the model providing the best fit [30]. Here, four models were compared where some parameters can be equal or vary across 2018 and 2019. In model 1, the same CFA is fit in every group. In model 2, the factor loadings are constrained to be equal across groups. In model 3, intercepts and factor loadings are constrained to be equal across groups. Finally, model 4 imposes a restriction where means, factor loadings and intercepts are set to be equal across groups. In 2019, our database included R scores, so that correlations between R score and MMI score could also be computed.

## Results

This study included 1438 candidates who did their IFMMI in 2018 (95.2% of the cohort) and 1440 candidates in 2019 (90.8% of the cohort) who gave written consent to participate. The mean age of participants was 21.0 years old in 2018 and 21.7 years old in 2019. Regarding gender, 886 (61.6%) of participants were female in 2018 and 888 (61.7%) in 2019. Descriptive statistics for each of the 10 MMI station scores are provided in Table 2. The overall mean adjusted scores was 65.24 for 2018 (min = 58.22, max = 70.43, sd = 3.38) and 70.46 for 2019 (min = 65.84, max = 73.68, sd = 2.42). The reliability of scores was estimated using Cronbach’s alpha (0.68 for 2018 and 0.71 for 2019) and McDonald’s omega (0.73 for 2018 and 0.76 for 2019). Correlations (Pearson’s r) coefficients among station scores ranged between 0.07 and 0.44 for 2018 and between 0.13 and 0.55 for 2019 (see Table 3). In addition, in 2019, the R score was available for 1207 applicants in the database and showed no correlation with the MMI score (r = − 0.023, *p* = 0.430). A very weak positive correlation was observed between the R score and the collaboration stations’ subscore (r = 0.031, *p* = 0.031) and a very weak negative correlation was observed between the discussion stations’ subscore and the R score (r = − 0.060, *p* = 0.036). No correlation was seen between role-play station scores and R score (r = − 0.018, *p* = 0.524). In 2019, we also had data for 755 admitted students to Quebec medical schools and their mean IFMMI score was significantly higher than non-admitted students (76.64 vs 65.46, *p* < 0.001). This difference was observable across all station subtype scores.

**Table 2:** Descriptive statistics of MMI scores for the 2018 and 2019 IFMMI cohorts

	Station	Format	Mean	Median	SD	Min^a^	Max^a^	Skew	Kurt
2018	S1	Discussion	66.90	68.32	19.35	−14.57	111.02	−0.52	0.61
	S2	Collaboration	68.35	69.25	18.14	−10.04	114.14	−0.66	0.95
	S3	Collaboration	66.01	68.16	19.52	−5.35	112.94	−0.53	0.41
	S4	Discussion	65.72	67.38	20.67	−6.31	114.40	−0.49	0.03
	S5	Role-play	58.22	62.06	25.70	−38.51	113.13	− 0.48	−0.13
	S6	Discussion	65.82	67.15	20.44	−13.43	111.22	−0.52	0.26
	S7	Role-play	62.08	61.52	23.28	−16.45	111.59	−0.45	−0.10
	S8	Discussion	65.38	66.72	20.81	−22.89	119.07	−0.50	0.19
	S9	Discussion	70.43	70.98	19.72	−4.18	121.68	−0.58	0.29
	S10	Role-play	63.43	67.99	23.33	−17.84	114.76	−0.51	0.00
	Discussion (overall)		66.85	67.54	12.28	9.93	98.49	−0.51	0.86
	Collaboration (overall)		67.18	68.38	15.98	−1.71	106.45	−0.60	0.74
	Role-play (overall)		61.25	62.25	16.92	−4.48	100.49	−0.38	0.02
2019	S1	Discussion	71.50	71.48	18.61	−10.55	117.78	−0.67	0.59
	S2	Collaboration	69.70	70.61	18.70	−5.72	115.33	−0.77	1.04
	S3	Collaboration	69.69	70.61	18.24	2.63	109.11	−0.76	0.72
	S4	Discussion	72.90	72.82	17.13	4.64	111.41	−0.54	0.24
	S5	Role-play	65.84	67.77	22.66	−13.76	114.19	−0.50	0.02
	S6	Discussion	72.79	72.44	19.07	3.14	112.57	−0.51	0.06
	S7	Role-play	70.43	69.14	19.97	−24.95	119.22	−0.67	0.51
	S8	Discussion	70.26	72.86	20.88	−8.05	117.00	−0.60	0.16
	S9	Discussion	73.68	73.29	18.91	−10.90	108.25	−0.64	0.29
	S10	Role-play	67.77	71.19	23.35	−29.98	115.49	−0.54	−0.18
	Discussion (overall)		72.23	73.20	11.62	27.83	101.40	−0.48	0.26
	Collaboration (overall)		69.69	71.10	16.26	3.24	111.21	−0.81	1.01
	Role-play (overall)		68.02	69.34	15.20	9.32	101.75	−0.49	0.19

*Abbreviations*: *SD* standard deviation, *Min* minimum score, *Max* maximum score, *Skew* Skewness, *Kurt* Kurtosis

^a^ Negative values and values >100 are explained by the adjusted score according to rater’s leniency or stringency

**Table 3:**
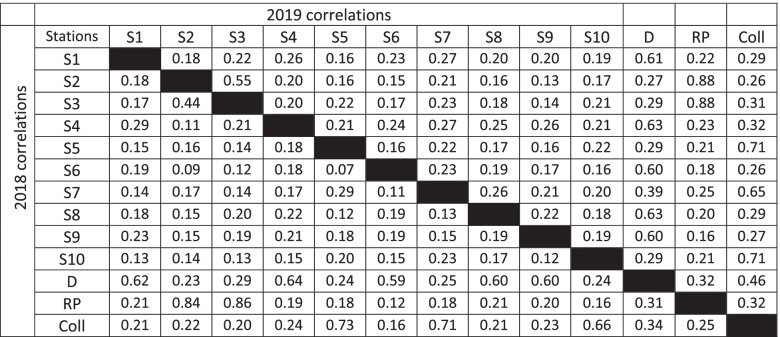
Pearson correlations between individual MMI stations’ scores for 2019 (upper triangle) and 2018 (lower triangle)

*D* discussion stations, *RP* role-play stations, *Coll* Collaboration stations

All correlations in the table are significant at *p* < 0.01

**Fig. 1:**
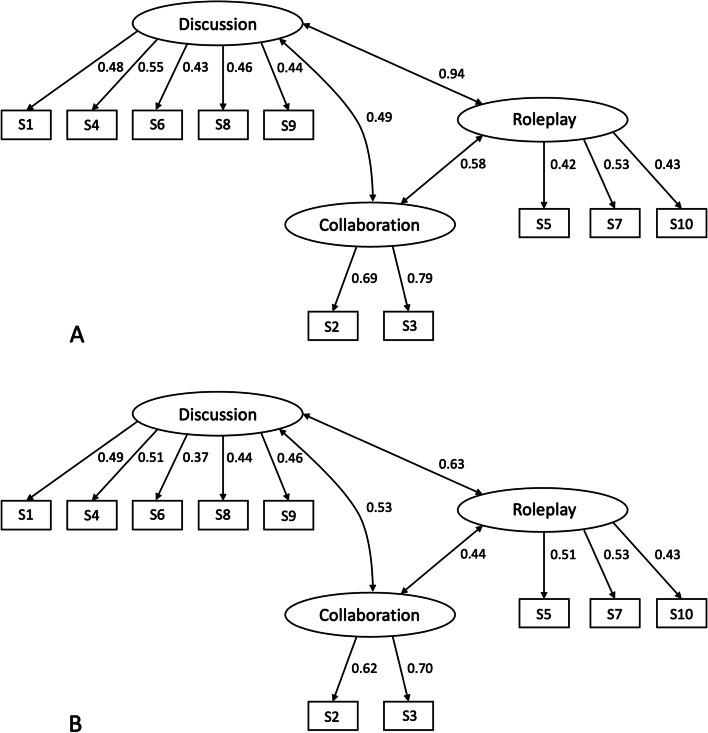
Standardized factor loadings and model covariates of the confirmatory factor analysis for 2018 (**A**) and 2019 (**B**). Stations are represented as rectangles and station formats are represented as ovals. Coefficients on the arrows can be interpreted as correlations

Results of the CFA are presented in Fig. 1A for the 2018 cohort and Fig. 1B for 2019. The analysis confirmed a three-factor solution: discussion stations, role-play stations and collaboration stations all loaded adequately with their scores. The model fit for 2018 was excellent with a CFI of 0.983, a TLI of 0.976, a SRMR of 0.021 and a RMSEA of 0.023. In comparison, the single factor solution presented lower fit values (CFI = 0.819, TLI = 0.767, SRMR = 0.049 and RMSEA = 0.070). This trend is similar for 2019. The model fit for the three-factor structure was also excellent (CFI = 0.99, TLI = 1.00, SRMR = 0.015 and RMSEA< 0.000) and clearly superior to the model with a single factor (CFI = 0.835, TLI = 0.788, SRMR = 0.050 and RMSEA = 0.077).

Results of the multigroup CFA are presented in Table 4. They show no substantial difference between 2018 and 2019. The fit measures are all excellent, even when comparing the fit of a progressively more constrained model. Model 1 presented the lower Akaike information criterion (AIC) value and Model 4 shows the lower Bayesian information criterion (BIC) value, suggesting that there is a very small difference between the various models according to the year.

**Table 4:** Multiple group confirmatory factor analysis based on year (2018 and 2019): model fit measures

	CFI	TLI	RMSEA	SRMR	AIC	BIC
Model 1	0.993	.992	0.014	0.017	241,213.843	241,604.664
Model 2	0.994	.990	0.016	0.017	241,219.843	241,628.429
Model 3	0.993	.970	0.015	0.019	241,214.457	241,581.592
Model 4	0.984	.982	0.021	0.024	241,230.547	241,538.467

*Abbreviations*: *CFI* Comparative fit index, *TLI* Tucker-Lewis index, *RMSEA* Root mean square error of approximation, *AIC* Akaike information criterion, *BIC* Bayesian information criterion

## Discussion

In this study of the 2018 and 2019 IFMMI cohorts, we found that the three different station formats (discussions, role-plays and collaboration) resulted in a three-factor structure that was consistent across 2 years. This suggests that, in our context, stations purposively designed differently are assessing different dimensions. We must, however, interpret with caution the three-factor structure – it may be related to different levels of task complexity, the different ways raters score the different tasks, or differences in underlying constructs. Some variations in the association between discussion and role-play station subscores also suggest that individual design choices are still important in the factor loading of these stations. Moreover, across all three station formats, the correlation with the R score was either absent or very weak, suggesting that all three station formats are assessing non-cognitive attributes. Overall, these results are comparable to the factor analyses recently performed by Renaud et al. on the 2010-2017 iterations of the IFMMI, that relied solely on discussion and role-play stations [17]. In this study, all multidimensional models considering station formats had a better fit than the unidimensional models [17].

So far, in other contexts, very few studies have looked at how different station formats may contribute to the dimensionality of an MMI. The study by Mirghani et al. [31] used EFA and could adequately differentiate stations that were intended to measure visuomotor skills and soft skills, where six stations involving mainly discussion and reflection loaded in one factor, and four stations involving manual dexterity or motor tasks loaded in different factors. Considering “soft skills” stations, a German study about MMI recently hypothesized that role-play and discussion stations were assessing different constructs, thus creating small but perceptible subgroup differences [32]. No factor analysis was performed, however, in this context. To our knowledge, the only CFA published on MMI in a different context was by Oliver et al. [9] and achieved a good fit (CFI of 0.94 and 0.97 for a one- and two-factor model, respectively). However, this study was comparing factors assessed on two scales (communication and problem evaluation) that were used across all stations. This differs significantly from our context where there was only one scale per station and precludes any comparison. In our case, CFA was performed according to a hypothesis-driven process based on station format, rather than scales, and is likely to provide more meaningful results than an EFA [33]. However, whether this factorial structure underpins different constructs or simply different tasks remains unclear, but it is likely that we are assessing different skills when using different station formats.

Although MMI are very often used across healthcare professions selection, the exact role and impact of using various station formats for MMI have not been extensively studied. From a theoretical perspective, changing station format is the equivalent of modifying what Lievens and Sackett would call “predictor method factors”, i.e. small components of the selection method [34]. Although the stimulus format (face-to-face) is the same in the three formats, various levels of contextualization or stimulus presentation consistency are expected from these three distinct tasks. For example, Eva et al. demonstrated in an experimental format that reliability could change according to the type of question asked (behavioral vs situational), which is a direct example of how modifying a small component of a selection tool can optimize results [35]. Research conducted on the IFMMI suggest that the predictive validity and reliability of both role-play and discussion stations are comparable [17]. However, it seems plausible that changing the station formats will have an impact on what they assess and we encourage researchers to carefully study the differential predictive validity of various station formats. For instance, a recent German study found that some subgroup differences (e.g. male/female) would vary according to station format, suggesting that the constructs assessed in these different formats were different [32]. The authors suggested that role-play (or “simulation”) stations required more affective empathy, as opposed to discussion or interview stations, that perhaps required more cognitive skills related to perspective taking or reflection [32]. Moreover, the fact that our third station format, collaborative stations, loaded in a different factor also suggests that they are purposively assessing a different dimension than the two other station formats, which is likely related to leadership and collaboration.

Although each station format seems to be assessing something different, the covariance between the three station formats remains moderate-to-high (0.44 to 0.63), suggesting that elements such as communication skills are likely to be assessed transversally. Also, although our three-factor structure does show differences between station formats, it does not provide any details as to which constructs are specifically assessed within each format, or within each individual station. Moreover, our observation may not be generalizable to other institutions, depending on the actual content of the MMI stations and implementation parameters. This is illustrated by the contradictory findings described so far in the literature regarding the dimensionality of various MMIs [36]. The use of factor analysis has been criticized in MMI-like assessments, because of its inability to account for the complexity of the design, including possible sources of variance related to the assessor, the candidate and the station [36, 37]. The ratings in the current study were, however, corrected for raters’ inconsistencies, possibly lowering this source of unwanted variance.

Finding a three-factor solution does have significant implications regarding reliability measurement. Indeed, if three different dimensions are measured in our MMI, few stations are assessing each one. This raises questions about the appropriateness of Cronbach’s alpha to estimate reliability. Recent commentaries reiterated its usefulness when the assessment tool is unidimensional, which is not the case here and possibly not the case in other MMIs relying on multiple station subtypes [38]. We therefore encourage MMI designers to perform a factor analysis as part of quality improvement and, if applicable, choose a reliability coefficient that can be applied to multiple dimensions, such as McDonald’s omega [39–41]. Furthermore, since the tool is multidimensional, it may become relevant to look at individual station scores, or specific station format scores, given that they each measure something different. For example, a “pass” mark could be implemented for each station format’s subscore, at least to ensure that applicants performed reasonably well in each of them.

## Conclusions

In this study, we demonstrated using CFA that the 2018 and 2019 iterations of the IFMMI had tridimensional structure that was explained by station formats (discussion, role-play, collaboration) and which was consistent across two cohorts. Our findings constitute an additional argument in the MMI validation process, along with previous observations about IFMMI reliability and their predictive validity with clerkship rotation performance. This process also informs how reliability will be measured for future iterations of the IFMMI. Further studies will need to assess if the different station formats have different psychometric properties, predictive values and subgroup differences in various contexts. Indeed, different station formats may be assessing different dimensions, but whether certain formats allow the selection of better-suited candidates is a completely different question.

## Data Availability

The datasets generated and/or analysed during the current study are not publicly available due to ethical reasons (including the protection of the confidentiality of applicant’s performance data for this high-stake selection interview) and their ownership by the medical schools. However, anonymized data may be available from the corresponding author on reasonable request conditionally to REB approval and approval by all the institutions involved in the data collection.

## Abbreviations

MMI: Multiple mini-interviews
AC: Assessment Centers
IFMMI: Integrated French MMI
CFA: Confirmatory factor analysis
CFI: Comparative fit index
TLI: Tucker Lewis index
SRMR: Standardized Root Mean Square Residual
RMSEA: Root Mean Square Error of Approximation
EFA: Exploratory factor analysis
GPA: Grade point average
AIC: Akaike information criterion
BIC: Bayesian information criterion
OSCE: Objective structured clinical examination
